# Colonic Carcinoma Masquerading as Scrotal Swelling: A Case Report and Review of Literature

**DOI:** 10.1100/tsw.2007.156

**Published:** 2007-04-30

**Authors:** Sharath C. V. Paravastu, Meenakshi Batra, Krishnan Ananthakrishnan

**Affiliations:** Department of Urology, Wrexham Maelor Hospital, Croesnewydd Road, Wrexham LL13 7TD, UK; Department of Pathology, Wrexham Maelor Hospital, Croesnewydd Road, Wrexham LL13 7TD, UK

**Keywords:** spermatic cord neoplasm, spermatic cord metastases, colon, adenocarcinoma

## Abstract

Tumours of the spermatic cord are rare. Most tumours of the spermatic cord are metastatic and are typically an incidental finding at orchidectomy for other pathology. Primary pathology is usually from the gastrointestinal tract. We report a very rare presentation of an asymptomatic gastrointestinal tumour as a spermatic cord mass.

male patient presented with a painless scrotal swelling. Radical orchidectomy revealed an adenocarcinoma in the spermatic cord. Further investigations disclosed an adenocarcinoma of the descending colon, metastasing to the spermatic cord that gave a false notion of a scrotal swelling secondary to infection. This unusual situation reminds us that spermatic cord metastases are rare. In the event of nonresponding scrotal swelling to antibiotics, further investigations would be prudent, whilst awaiting the definitive pathology report from the radical orchidectomy.

Tumours of the spermatic cord are rare and mostly mesenchymal in origin[1]. Even rarer are tumours of epithelial origin (9%)[2]. Almost all epithelial tumours involving the spermatic cord are metastatic[1] and are typically an incidental finding at orchidectomy for other pathology (testicular cancers or prostate cancers). Metastasis to the spermatic cord and/or the intrascrotal contents is generally found in conjunction with widespread advanced disease. Primary sites for these tumours include stomach, prostate, kidney, colon, ileum, appendix, and pancreas. Metastatic neoplasms from the digestive tract occur less frequently, and for a metastasis to be the first manifestation of a primary tumour is even more atypical[3]. These conditions present a diagnostic and therapeutic conundrum[4,5]. We describe a case of descending colon adenocarcinoma metastasing to the spermatic cord, whose initial presentation was painless scrotal swelling.

## CASE PRESENTATION

### Case Report

A 62-year-old, fit man was referred with a 2-month history of painless left scrotal swelling. He denied trauma, previous similar episodes, and change in bowel habits, abdominal pain, or any systemic upset. On examination, an enlarged epididymis with extension up to the spermatic cord was noted, consistent with the clinical diagnosis of epididymitis. The testis was normal in consistency and size. Urine dipstick was negative. Routine blood tests, including the tumour markers (α-fetoprotein, β-HCG, PLAP) were normal, but LDH was raised. Ultrasonography illustrated an epididymal swelling with an extension up the spermatic cord, in keeping with inflammatory aetiology (Fig. 1). Further review 1 month later showed the inflammatory mass to have increased in size despite the 4-week course of antibiotics.

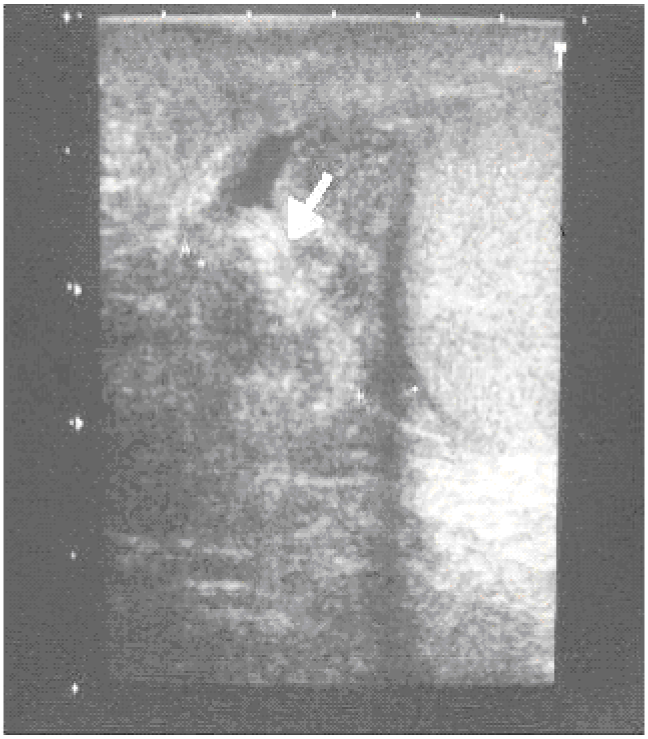

**FIGURE 1.** Ultrasound demonstrating epididymal swelling consistent with inflammation.

## Management

The patient underwent radical inguinal orchidectomy. Macroscopic appearances at surgery suggested epididymitis involving the spermatic cord. However, histology sections from the spermatic cord revealed extensive infiltration by atypical glandular structures. The epididymis and testes were reported normal. Immunohistochemical staining for prostate-specific antigen was negative. Pathological diagnosis was metastatic adenocarcinoma with extensive vascular permeation involving the spermatic cord with likely primary pathology from the gastrointestinal tract (Fig. 2).

CT demonstrated multiple liver, mediastinal, and lung metastases, along with abnormal-looking, thickened, descending colon (Fig. 3). Serum CEA was elevated to 167 µg/l. Colonoscopy corroborated a stenosing lesion in the descending colon and the biopsy verified the histology of poorly differentiated adenocarcinoma. Notwithstanding this metastatic presentation, the patient was completely asymptomatic with a very good performance status. He completed palliative chemotherapy with irinotecan, fluorouracil, and cetuximab with a good partial response. CEA came down to 6.7 µg/l and the CT scans demonstrated a decrease in the metastatic tumour burden, especially in the liver and mediastinum. He remains clinically well 18 months following the diagnosis.

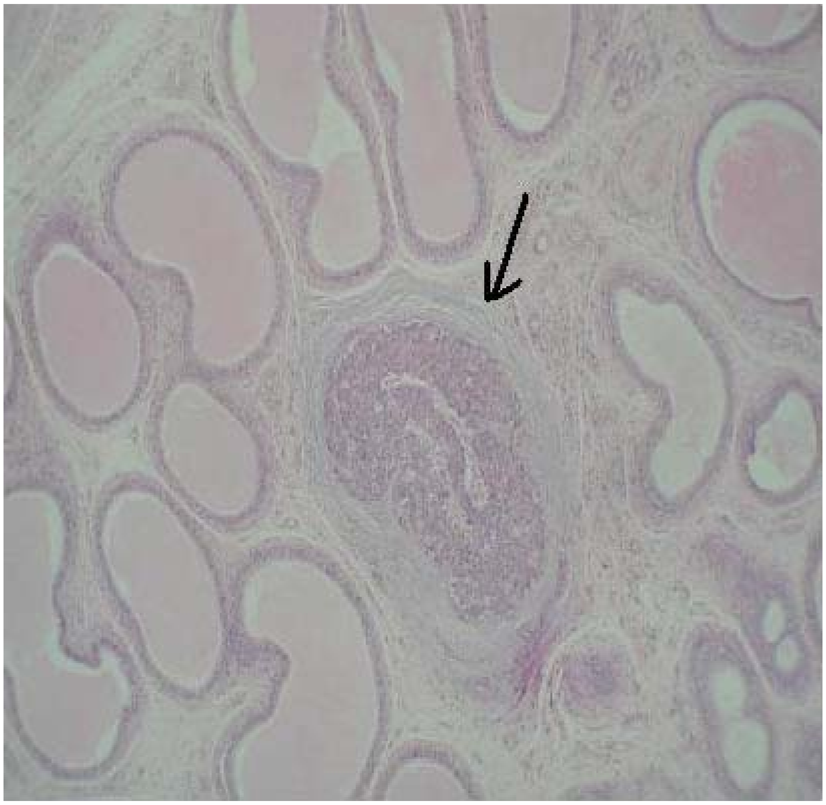

**FIGURE 2.** Histology slide showing metastatic change in the epididymis (shown with black arrow).

## DISCUSSION

In a review by Beccia et al., 31% of 636 tumours of the spermatic cord were malignant, of which 91% were sarcomas[2]. Blitzer et al. studied 201 malignant primary tumours of the spermatic cord and found only four unspecified carcinomas, thus confirming the view that almost all epithelial tumours of the spermatic cord are of metastatic origin[6].

The potential patterns of metastatic tumour spread to the spermatic cord and paratesticular tissues include retrograde venous extension, arterial emboli, retrograde spread by way of the vas deferens, and lymphatic spread[7]. Of these, metastatic spread exclusively by vas deferens is doubtful since epididymal metastases have been reported in men with carcinoma prostate who have had vasectomy[8]. Rarely, a patent processus vaginalis may lead to metastatic spread of malignant mesothelioma to the spermatic cord[9]. Retrograde lymphatic spread through retroperitoneal lymph nodes has been demonstrated as the most probable mechanism of extension seen in gastrointestinal tumours. In contrast to previous studies, in our patient, haematogenous spread is the most likely route, supported by the presence of extensive vascular invasion by the tumour cells with minimal stromal invasion in epididymis and widespread lung and liver metastasis.

Metastasis to the spermatic cord is rare. Metastasis from the gastrointestinal tract is infrequent and for a metastasis to be the first manifestation of a primary tumour is even more unusual[3]. Only 9.5% of metastasis appears as the first sign of an occult neoplasm and in 47.6% of these cases, the metastasis and the primary tumour are found simultaneously[10]. Although the histological types of spermatic cord and epididymal metastasis cover a wide variety, carcinomas of the gastrointestinal tract (stomach, pancreas, colon, caecum, jejunum, bile duct, and appendix) and prostate are the most frequent, together accounting for more than one-half of the cases[5]. Other tumours in which metastasis to the spermatic cord has been reported include carcinomas of the kidney, ureter, and bladder[11]. The liver is the most common site of

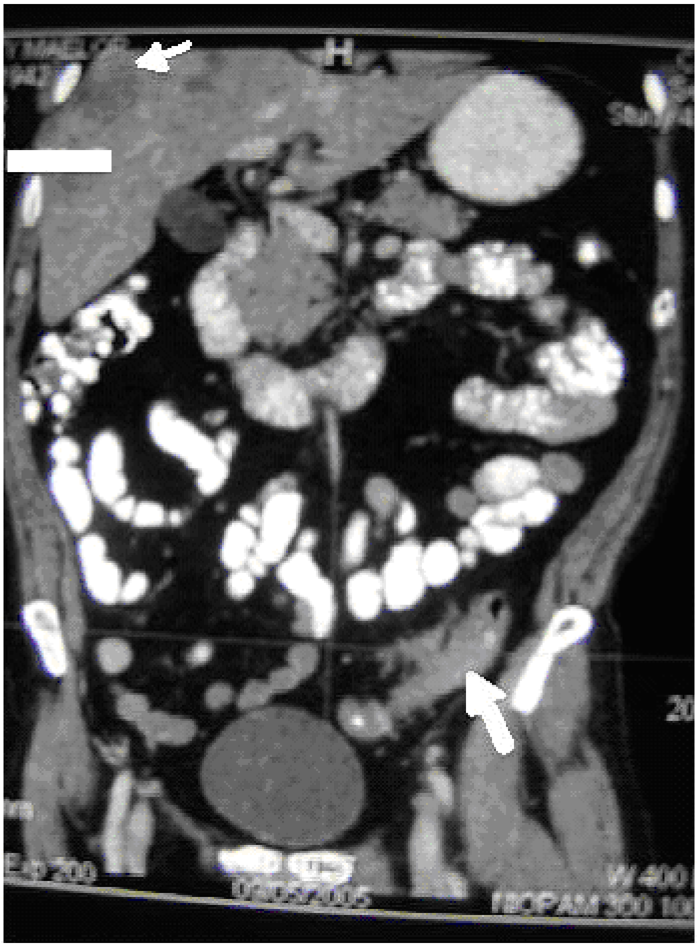

**FIGURE 3.** CT abdomen showing thickened, descending colon with extensive liver metastasis (both shown in arrows).

haematogenous dissemination of colorectal cancer, followed by the lungs and bones. Less commonly reported sites include the brain, adrenals, and skin. The spermatic cord and epididymis are extremely rare sites for metastatic tumour spread[2].

The reasons for the relatively low incidence of metastases to this site are not clear and one may speculate that the relatively low temperatures in this region are not conducive to the establishment of metastatic tumour cells[12]. The prognosis is generally poor, with the average survival period from the time of diagnosis of the metastasis being only 9.1 months[10].

## CONCLUSION

This case report highlights the need to consider the diagnosis of primary or secondary spermatic cord tumour when an apparent scrotal mass does not resolve with antibiotics. In ambiguous testicular and paratesticular masses (especially if the patient is past the usual age for a primary testicular tumour), and in the setting of a hydrocoele, or nonspecific systemic symptoms, an inguinal surgical exploration is warranted.

## ACKNOWLEDGEMENTS

The authors would like to thank Mr. Alan DeBolla and Dr. Anthony Burdge for their intellectual input in the writing of this manuscript. Written consent was obtained from the patient for publication of this report.

## References

1. Mostofi, F.K. and Price, E.B., Jr. (1973) Tumours of the male genital system. In *Atlas of Tumor Pathology*. Second Series. Fascicle 8. Armed Force Institute of Pathology, Washington, D.C. pp. 143–173.
2. Beccia, D.J., Krane, R.J., and Olsson, C.A. (1976) Clinical management of non-testicular intrascrotal tumours. *J. Urol*. **116**, 476–479.
3. Mcacham, R.B., Mata, J.A., Espada, R., Wheeler, T.M., Schum, C.W., and Scardino, P.T. (1988) Testicular metastasis as the first manifestation of colon carcinoma. *J. Urol*. **140**, 621–622.
4. Lioe, T.F. and Biggart, J.D. (1993) Tumours of the spermatic cord and paratesticular tissue. A clinicopathological study. *Br. J. Urol*. **71**, 600–606.
5. Polychronidis, A., Tsolos, C., Sivridis, E., Botaitis, S., and Simopoulos, C. (2002) Spermatic cord metastasis as an initial manifestation of sigmoid colon carcinoma: report of a case. *Surg. Today* **32(4),** 376–377.
6. Blitzer, P.H., Dosoretz, D.E., Proppe, K.H., and Shipley, W.U. (1981) Treatment of malignant tumours of the spermatic cord: a study of 10 cases and a review of the literature. *J. Urol*. **126**, 611–614.
7. Monn, L. and Poticha, S.M. (1975) Metastatic tumours of spermatic cord. *Urology* **5(6),** 821–823.
8. Talbot, R.W. and McCann, B.G. (1979) Secondary prostatic tumour of the spermatic cord and epididymis 5 years after prostatectomy and vasectomy. *Br. J. Urol*. **51**, 48.
9. Lieber, C., Katzenwadel, A., Popken, G., Kersten, A., and Schultze-Seemann, W. (2000) Tumour of the spermatic cord: an unusual primary manifestation of an epithelial mesothelioma of the peritoneum with patent processus vaginalis. *BJU Int*. **86**, 142–143.
10. Algaba, F., Santaularia, J.M., and Villavicencio, H. (1983) Metastatic tumour of the epididymis and spermatic cord. *Eur. Urol*. **9**, 56–59.
11. Issa, M.M., Kabalin, J.N., Dietrick, D.D., Reese, J., and Freiha, F.S. (1994) Spermatic cord metastasis from transitional cell carcinoma. *Urology* **43(4),** 561–563.
12. Smallman, L.A. and Odera, J.K. (1984) Primary carcinoma of sigmoid colon metastasising to epididymis. *Urology* **23**, 598–599.
